# Comment on Giuseppe Genchi et al. Mercury Exposure and Heart Diseases. *Int. J. Environ. Res. Public Health* 2017, *14*, 74

**DOI:** 10.3390/ijerph14070733

**Published:** 2017-07-06

**Authors:** S. M. J. Mortazavi, Ghazal Mortazavi, Maryam Paknahad

**Affiliations:** 1 Diagnostic Imaging Center, Fox Chase Cancer Center, Philadelphia 19111, PA, USA; S.M.Javad.Mortazavi@fccc.edu; 2 Ionizing and Non-Ionizing Radiation Protection Research Center (INIRPRC), Shiraz University of Medical Sciences, Shiraz 7134845794, Iran; mortazavismj@gmail.com; 3 Oral and Maxillofacial Radiology Department, School of Dentistry, Shiraz University of Medical Sciences, Shiraz 713451836, Iran

Cenchi et al. [1] have recently published an article entitled “Mercury Exposure and Heart Diseases” that is published in *Int. J. Environ. Res. Public Health* 2017, 14, 74; doi:10.3390/ijerph14010074. In this article, the authors reviewed the toxicity of mercury and focused on the toxic effects on the cardiovascular system. Although the paper authored by Cenchi et al. is a well-structured article which can be considered a significant contribution in this field, it has some shortcomings. Over the past several years, we have shown that when dental amalgam fillings are exposed to electromagnetic fields (EMFs) produced by magnetic resonance imaging (MRI) or those generated by other sources such as mobile phones, the level of mercury released from dental amalgam restorations can be significantly increased [2,3]. It is worth noting that rapidly increasing advances in modern technologies such as telecommunication and the exponential rise in the use of wireless systems have drastically increased the human exposure to different sources of EMFs (mobile phones, mobile base stations, cordless phones, Wi-Fi routers, radio and TV broadcasting, etc.). Studies performed on microleakage of amalgam have further confirmed the findings obtained in our studies on EMF-induced accelerated mercury release [4,5]. Furthermore, Kursun et al. have also demonstrated that exposure to X-rays (an energetic part of the electromagnetic radiation spectrum) can increase the mercury release from amalgam fillings [6]. Although the mercury levels which normally can be released from amalgam fillings, even in the presence of EMFs, are not high enough to cause toxicity, a hypersensitive subpopulation, pregnant women, and children may be affected by this phenomenon. These findings have recently been reviewed by Mortazavi and Mortazavi [7]. Based on the substantial evidence provided above, a shortcoming of this review conducted by Cenchi et al. [1] comes from this point that these researchers have not considered the substantial evidence which indicates the role of exposure to EMFs on the release of mercury from dental amalgam restorations.
